# Impact of a Brief Family Skills Training Programme “Strong Families” in a Low–Middle-Income-Country: Cambodia

**DOI:** 10.3390/children12060728

**Published:** 2025-05-31

**Authors:** Aala El-Khani, Dina Idriss-Wheeler, Santana Chea, Shatha Darwish, Wadih Maalouf

**Affiliations:** 1 United Nations Office on Drugs and Crime (UNODC), Prevention, Treatment and Rehabilitation Section, Drug Prevention and Health Branch, Division of Operations, Wagramer Strasse 5, A-1400 Vienna, Austria; wadih.maalouf@un.org; 2 Division of Psychology and Mental Health, The University of Manchester, Manchester M13 9PL, UK; 3 Faculty of Health Sciences, Population Health, University of Ottawa, Ottawa, ON K1N 6N5, Canada; didri040@uottawa.ca; 4 Live and Learn Cambodia, Phnom Penh 12302, Cambodia; cambodia.office@livelearn.org; 5 Department of Psychology, Faculty of Health and Education, Manchester Metropolitan University, Manchester M15 6BX, UK; 23662154@stu.mmu.ac.uk

**Keywords:** parenting skills, Cambodia, Strong Families programme, resilience, mental health

## Abstract

**Introduction:** Children living in low- and middle-income countries (LMICs) are at increased risk of emotional and behavioural challenges, often linked to caregiver stress and harsh parenting practices. Strengthening family functioning through parenting interventions is a critical strategy for improving child mental health in these settings. The Strong Families programme was developed as a light-touch family skills intervention for high-stress, low-resource environments. **Methods:** A multisite pilot feasibility and acceptability study was conducted in Cambodia with 40 families. Caregivers and children (aged 8–15) participated in a 3-week intervention, with one session per week. Data were collected using the Parenting and Family Adjustment Scales (PAFAS), the Strengths and Difficulties Questionnaire (SDQ), and the Child and Youth Resilience Measure (CYRM-R) at baseline, two weeks, and six weeks post-intervention. Repeated measures ANOVA and Friedman’s ANOVA were used to assess changes over time. **Results:** Caregivers showed statistically significant improvements across all PAFAS subscales. For example, coercive parenting scores decreased from 8.13 at baseline to 4.00 post-intervention and 2.33 at follow-up (F(2,78) = 59.76, *p* < 0.001). Positive encouragement improved from 2.60 to 1.00 and 0.33, respectively (F(2,78) = 27.05, *p* < 0.001). In terms of child outcomes, SDQ total difficulty scores declined from 20.68 to 16.55 over time (F(2,78) = 7.58, *p* = 0.001). Emotional problems dropped from 5.60 to 2.38 (χ^2^(2) = 21.17, *p* < 0.001), and conduct problems from 4.33 to 2.68 (F(2,78) = 11.35, *p* < 0.001). Prosocial behaviours increased from 5.60 to 9.45 (F(2,78) = 69.93, *p* < 0.001). Personal resilience scores rose from 32.70 at baseline to 47.48 at follow-up (χ^2^(2) = 62.42, *p* < 0.001), while caregiver resilience improved from 23.63 to 33.63 (χ^2^(2) = 61.83, *p* < 0.001). Improvements were particularly pronounced among families with the highest baseline challenges. **Conclusions:** Findings indicate that the Strong Families programme is feasible and effective in improving parenting skills, family adjustment, child mental health, and resilience in a Cambodian LMIC context. These results reinforce the programme’s potential for integration into broader national strategies to improve psychosocial outcomes for families in high-stress, low-resource environments.

## 1. Introduction

### 1.1. Child Well-Being and Caregiver Support in Low-Resource Settings

Prevalence studies indicate that children who are raised in low- and middle-income countries (LMICs) experience high rates of violence within their families in the form of psychological or physical punishment [1]. In the East Asia and Pacific region, parents or primary caregivers are the most commonly identified perpetrators of the violence, with 63% of girls having experienced such abuse by their mothers in Cambodia, 28% by their fathers in the Philippines, and 63% by their mothers in Timor-Leste [2]. Stressed caregivers, associated with living conditions in LMIC, are less likely to provide children with essential positive interactions that are key to promoting healthy psychosocial and physical development [3]. Instead, children in LMIC are more likely to experience harsh parenting, which increases their risk of a variety of emotional and behavioural problems [3,4]. This in turn compounds the vulnerabilities children living in challenged settings face, including mental health and behavioural challenges [5,6].

The World Health Organization (WHO) defines good mental health as “a state of well being, in which the individual realises his or her own abilities, can cope with the normal stresses of life, can work productively and fruitfully, and is able to make a contribution to his or her community” [7,8]. During prolonged periods of stress, children are more likely to develop unaddressed vulnerabilities associated with poor health and developmental outcomes, such as poor mental health, violence, lower educational achievement, and substance use [9,10]. The primary caregivers of children and youth can have a vital role in the protection of children’s mental health in challenging, stressful contexts [11], as well as the prevention of psychological morbidity [12,13,14]. When considering those families residing in challenged settings, these parental and family factors are even more central for children to achieve positive outcomes [8,15]. Even in families experiencing chronic adversity, strong parent–child relationships and consistent parenting can help mitigate the impact of risk factors on child development, providing both protection and support [2,16].

### 1.2. Family Skills Programmes

Caregivers and their children living in LMIC often face severe and prolonged stress and adversity, along with poverty, and may struggle to provide appropriate parenting. This can significantly impact the family dynamics and subsequent child outcomes [17,18]. Therefore, the relationship caregivers have with their children can act as powerful risk or protective factors, either further compounding or mitigating the impacts of living through challenging contexts on children.

Family skills programmes that encourage safe and nurturing relationships between primary caregivers and their children can successfully prevent a multitude of negative social outcomes (including drug use, child maltreatment, and poor mental health) and significantly reduce childhood aggression [19]. Such tools provide caregivers with the basic knowledge to apply positive parenting skills that allow parents to cope and adapt to the different challenges that arise with parenting children. A key component of such interventions is the opportunity for caregivers to practice these skills through competency enhancement and support [20]. These primary prevention programmes focus on enhancing the bond between caregivers and their children by strengthening parenting skills that foster key protective family factors, such as effective communication, trust-building, problem-solving, and conflict resolution.

The UNODC-WHO International Standards on Drug Use Prevention [21], INSPIRE initiative to end violence against children [22], WHO-led Violence Prevention Alliance [19,23], and WHO/UNICEF Helping Adolescents Thrive initiative [8] have all listed and recommended evidence-based family or parenting skills programmes, as a key factor that works to serve multi-outcome initiatives. A growing body of evidence demonstrates the effectiveness of family skills interventions in high-income and stable environments [24,25]. However, research on their application in lower-income and crisis-affected settings remains an area of ongoing development [26]. Systematic reviews highlight that international guidance for LMICs has often prioritised parenting-focused interventions, with limited synthesis available on family-based approaches that consider the broader family system [27,28]. Consequently, the United Nations Office on Drugs and Crime (UNODC) has launched a global prevention initiative, piloting and testing evidence-based family skills programmes in LMICs [29]. This initiative led to the development of various initiatives, including the Strong Families programme, a targeted, brief family skills intervention designed to enhance parenting practices, strengthen family resilience [30], and promote child well-being and mental health within the family unit.

The Strong Families programme is a family skills intervention designed for caregivers and children (ages 8–15) living in low-resource and high-stress settings, including LMICs and humanitarian contexts. The programme aims to strengthen family resilience by helping families recognise and build on their strengths, fostering positive communication, and improving coping mechanisms. It is grounded in three key theoretical frameworks: the biopsychosocial vulnerability model, which highlights the role of family coping strategies in mitigating conflict-related stress; the resiliency model, which underscores the importance of caregiver support in fostering children’s ability to adapt to adversity; and social learning theory, which emphasises the influence of caregiver–child interactions on social development. Through structured sessions incorporating role plays and interactive activities, the programme equips families with essential skills for emotional regulation, conflict resolution, and stress management. For a more detailed discussion on the Strong Families programme, including its theoretical foundations and implementation, please refer to our previously published paper by Haar et al. (2021) [30].

The Strong Families programme has been introduced in more than thirty countries. Findings from randomised controlled trials (RCTs) and single-arm implementation studies conducted in Iran [30], Afghanistan [31], as well as Serbia [32] and suggest that the programme is both feasible and adaptable for delivery in low-resource and high-stress environments. Additionally, evidence indicates that it can be effectively implemented by trained lay facilitators.

### 1.3. Aims and Objectives

The current study evaluated the feasibility, acceptability, and efficacy of the Strong Families programme for caregivers and their children aged 8 to 15 years in Cambodia. The goal was to improve family skills outcomes and the mental health of caregivers and children, as reported by the caregivers. Rigorously evaluating the effectiveness of the Strong Families programme in Cambodia addresses a critical evidence gap regarding its scalability and adaptability. Evaluating the programme in a new setting will not only validate or refine the existing theory of change but also provide essential insights into the contextual moderators of programme outcomes. This is particularly important for informing policy decisions related to national roll-out, adaptation requirements, and resource allocation.

**Figure 1:**
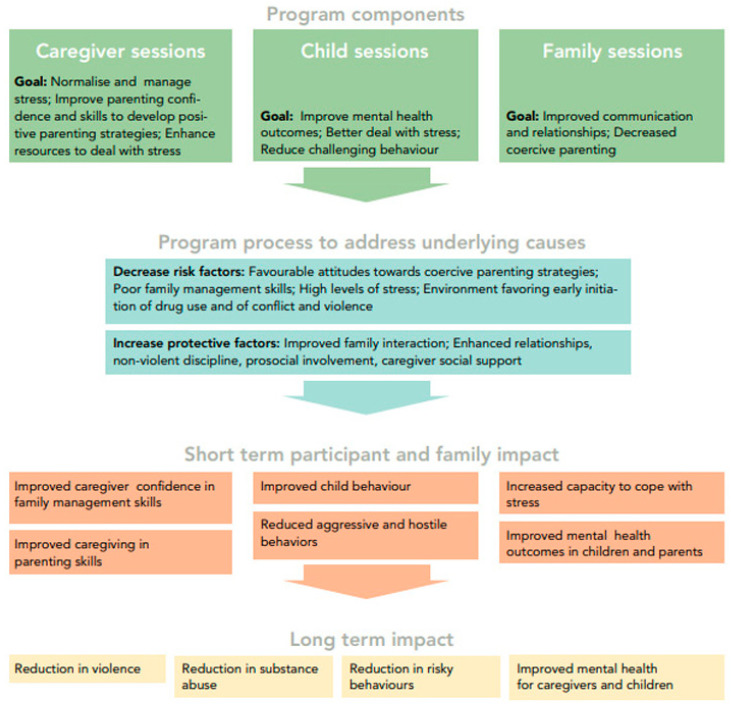
Logic framework of the Strong Families programme.

This study examined the programme’s impact across several dimensions outlined in the logical framework (Figure 1), including child mental health, parenting skills, family adjustment, and child resilience. Additionally, a sub-analysis focused on the most at-risk families identified at baseline to better understand the programme’s effects on these particular groups. The secondary objective was to assess the replicability of our previous single-arm pilot study in another country, Cambodia.

### 1.4. Country Context

Cambodia, an LMIC, is experiencing rapid economic and social transformation as it continues to recover from decades of conflict and transitions toward more democratic governance [33]. While the country has made significant strides in economic growth and poverty reduction, it remains largely rural. Its economic status has several impacts, including overall levels of education and low literacy levels [34,35]. Among Cambodia’s ethnolinguistic diversity are Cambodian perspectives on mental health and illness have been significantly shaped by exposure to the Khmer Rouge genocide, with mental illness often viewed as intrinsically linked to this historical trauma [33,36].

Violence against children remains prevalent in Cambodia, with over half of children reporting experiencing at least one violent incident before turning 18. Additionally, more than one-third of children aged 13 to 17 have witnessed physical violence in their homes within the past year [37,38]. A separate study involving 993 male and 950 female junior high school students from both urban and rural areas found that 27.9% of male students and 21.5% of female students had experienced domestic violence at least once. Furthermore, 18.0% of male students and 5.8% of female students reported experiencing violence within their community [37,39].

High levels of domestic violence also impact Cambodian children’s mental health, with a study showing that Cambodian children raised in a family where a parent/caregiver was physically hurt by another family member show symptoms of depression. Furthermore, they are likely to internalise and externalise violent behaviour in adolescence [40]. Findings from a study exploring intergenerational transmission of trauma in Cambodia suggest that parents and grandparents may use their experiences, beliefs, and attitudes to discipline their children in the process, shaping their belief system on health, trauma, and disabilities [41].

There is a significant lack of studies exploring parenting and family skills interventions in Cambodia. A recent systematic review and meta-analysis exploring the effectiveness of parenting interventions in preventing violence against children in LMIC, specifically East and Southeast Asia [2], identified 11 studies, though none from Cambodia. The results suggested that parenting interventions in this region can reduce rates of particular forms of violence against children, as well as promote positive parent–child interactions.

## 2. Materials and Methods

### 2.1. Programme Intervention

The Strong Families programme is a structured group intervention designed for children aged 8 to 15 and their primary caregivers, delivered over three weeks with one session per week. Each session includes up to 12 families, with a maximum of two caregivers and two children per family. The programme consists of both separate and joint sessions, where caregivers and children engage in targeted activities before coming together for a family session. In the first week, caregivers attend a one-hour pre-session focusing on normalising parenting challenges and learning stress management techniques. In weeks two and three, caregivers and children participate in separate one-hour sessions, followed by a one-hour family session. Caregivers practice using love and limits in their interactions, improving communication, and reinforcing positive behaviours, while children explore stress management, goal setting, and family responsibilities. The final family session emphasises strengthening family values and expressing appreciation for one another. For further details on the Strong Families programme intervention and its implementation, refer to Haar et al. (2021) [30]. For the Cambodian context, cultural adaptations were made through technical consultations with NGOs and UNODC, as well as advocacy meetings with national stakeholders responsible for drug use prevention, as well as family skills trainers to facilitate political endorsement. Training materials and evaluation tools were translated into Khmer to ensure accessibility and cultural relevance.

### 2.2. Trial Design, Sampling, Eligibility Criteria, and Sample Size

To evaluate the programme, an open, multisite pilot feasibility and acceptability trial was conducted. We prospectively collected the outcome data, assessing changes in parenting skills and family adjustment in caregivers, children’s behaviour, and children’s resilience capacities. The study employed a broad, non-targeted sampling strategy, where research assistants recruited families from the general population without focusing on any specific risk group. Participants were not assessed for clinical diagnoses; however, during the initial session, caregivers received informational leaflets detailing available resources for addressing severe stress reactions or any concerns related to physical, mental, or sexual health for themselves or their children. To be eligible for participation, families needed to speak Khmer, commit to attending the entire programme, and be in town and available for all study sessions and follow-up assessments. Exclusion criteria included prior participation in any family skills training programme within the past 24 months or cases where the caregiver and child did not reside together. Overall, 40 families were invited and participated in this single-arm study.

### 2.3. Procedure

The study was implemented in the Siem Pang district of Stung Treng Province in Cambodia. Participants were from 16 villages of 4 communes (Santepheap, Thmar Keo, Srae Sambor, and Praek Meas). These sites were selected by Live and Learn Cambodia, as they had strong links with the local authorities in these areas. Additionally, the community has been impacted by domestic violence.

Facilitators were selected based on their previous experience with prevention activities. Most of the facilitators nominated by local authorities had no previous experience facilitating prevention programmes. In September 2022, 20 facilitators (13 females and 7 males) were trained on the Strong Families programme in Cambodia by two trainers. In addition, 2 research assistants were trained by international UNODC staff on recruitment of participants, data collection, and acquaintance with the data collection tools. Following facilitator training, research assistants distributed brochures containing information to the caregivers of all children aged 8 to 15 years within the reach of their respective centres. Caregivers were also invited via a self-referral process to attend an information session, where they were given further verbal and written information, and questions were answered. Once families agreed to participate in the study, they attended an initial baseline measurement session, during which written informed consent was obtained before data collection began. Children provided assent, while caregivers signed consent forms. These documents confirmed that participants had the opportunity to ask questions about the study, participated voluntarily without any pressure, and understood that their data would remain anonymous and be used for scientific publications. Families in the study took part in activities between November 2022 and February 2023.

### 2.4. Data Collection

Data on family demographics, emotional and behavioural difficulties of children, parental skills, and family adjustment measures were collected through self-administered questionnaires completed by caregivers. Meanwhile, the social–ecological resilience was self-reported through children. The Family Demographic Questionnaire (FDQ) was completed one week before the intervention (t1) to gather baseline characteristics. Outcome measures, including the paper-based Parenting and Family Adjustment Scales (PAFAS), the Strengths and Difficulties Questionnaire (SDQ), and the Child and Youth Resilience Measure (CYRM-R), were initially collected at t1. These measures were subsequently reassessed at two (t2) and six weeks (t3) post-intervention to track changes and impacts.

#### 2.4.1. Family Demographic Questionnaire (FDQ)

The standardised FDQ had previously been applied in various country settings, including Iran [30], Afghanistan [31], Uzbekistan, Zanzibar, Serbia [32], the Philippines, and the Dominican Republic, with only minor modifications made to adapt it to the Cambodian context. The collection of demographic data aimed to enable stratified analyses, aligning with the study’s objectives. Additionally, the FDQ data was used to ensure a representative sample by comparing baseline characteristics such as caregiver age and gender, marital status, education level (both caregiver and partner), employment status, country of origin, duration of residence in Cambodia, number of children in the household, and the participating child’s age, gender, and relationship to the caregiver.

#### 2.4.2. Parenting and Family Adjustment Scales (PAFAS)

The Parenting and Family Adjustment Scales (PAFAS) [42] is a 30-item questionnaire designed to assess parenting practices and family functioning, which serve as key risk or protective factors for children’s emotional and behavioural well-being. It comprises two main scales: one evaluating parenting skills and parent–child relationships, and the other measuring family adjustment, including parental emotional well-being and family cohesion. Responses are rated on a three-point scale, with higher scores reflecting greater difficulties in parenting and family adjustment. The measure has demonstrated strong reliability and validity across diverse cultural settings, including Australia, Panama, China, and Arabic-speaking families in conflict-affected regions. While no established clinical cut-offs exist, for analytical purposes, families scoring in the highest 75th percentile at baseline were categorised as “most-at-risk families”. This study used PAFAS to evaluate short-term caregiver outcomes, including improved confidence in family management, improved parenting skills, and increased capacity to cope with stress. For further details, refer to the published study by Haar et al. (2021) [30].

#### 2.4.3. Strengths and Difficulties Questionnaire (SDQ)

The SDQ is widely used, and available in 45 languages, to evaluate children’s emotional, social, and behavioural difficulties within the past six months. It consists of 25 items, such as “I try to be nice to other people. I care about their feelings”, “I am often unhappy, downhearted or tearful”, “I take things that are not mine from home, school or elsewhere”, and “I fight a lot. I can make other people do what I want”. Responses are rated on a three-point scale from 0 (“Not True”) to 2 (“Certainly True”), with some items reverse-scored. The questionnaire is structured into five subscales that measure emotional difficulties, conduct issues, hyperactivity, peer relationship challenges, and prosocial behaviours. A total difficulties score is derived by summing four of the subscales, excluding prosocial behaviours, with possible scores ranging from 0 to 40, where higher scores indicate greater levels of behavioural and emotional difficulties [43].

The SDQ is frequently used in family skills research to assess changes before and after an intervention, as well as in both short- and long-term follow-ups [30,31,32]. For this study, we applied the Khmer translation and utilised the four-band categorisation system to classify SDQ scores into “close to average”, “slightly raised/lowered”, “high/low”, and “very high/low” risk [44]. The SDQ was used to evaluate potential shifts in children’s outcomes, focusing on short-term effects such as “Improved child behaviour”, “Reduced aggressive and hostile behaviour”, and “Enhanced mental health in children”, as outlined in the logical framework (Figure 1).

#### 2.4.4. Child and Youth Resilience Measure (CYRM-R)

The CYRM-R is a self-report tool designed to assess social–ecological resilience and has been translated into over 20 languages [45]. It is particularly effective for epidemiological research when paired with measures of psychosocial stress and mental health difficulties, such as the SDQ [46]. In alignment with the logic model of the Strong Families programme [39], this study used the CYRM-R to evaluate short-term impacts, including “Reduced aggressive and hostile behaviors”, “Increased capacity to cope with stress”, and “Improved mental health outcomes in children”, which contribute to long-term resilience outcomes [47].

The CYRM-R consists of 17 items, with 3- and 5-point Likert scale versions tailored for different age groups. This study employed the 5-point child version (for ages 5–9), incorporating questions like “I talk to my family/caregiver(s) about how I feel”, “I feel supported by my friends”, and “People like to spend time with me”, rated from 1 (“Not at all”) to 5 (“A lot”) [48]. For this study, the tool was translated and refined by local researchers to ensure cultural and linguistic appropriateness in Cambodia. This was through two steps, firstly translation from English to the local language by one team of researchers, and then back translated to English by another team of researchers, then compared for accuracy. The overall resilience score ranges from 17 to 85, derived from two subscales: personal resilience (10–50 points) and caregiver resilience (7–35 points), with higher scores indicating greater resilience. Given that resilience is context-dependent, no standardised cut-off scores have been established [45]. However, following previous recommendations, children scoring in the lowest third (33rd percentile) at baseline were categorised as “most-at-risk families” in the analysis.

### 2.5. Statistical Analysis

Quantitative data that was collected through and housed in EpiData was downloaded to an Excel spreadsheet and analysed using STATA (version 18; StataCorp, College Station, TX, USA). Raw data cleaning included completeness checks and outlier observations. There were no missing data found; however, detected outliers and data errors were checked, verified, and corrected by a community outreach specialist who oversaw data collection. For the FDQ data, descriptive analyses were performed on the continuous variables, including the calculation of the mean, standard deviation (SD), and range of scores (minimum and maximum). Bivariate analyses, utilising *t*-tests for normally distributed data and Mann–Whitney U tests for non-normal distributions, were conducted to identify demographic differences within the sample. For categorical variables, chi-square tests were employed to examine differences in frequency or proportion across categories. The threshold for statistical significance was established at a *p*-value lower than 0.05, with 95% confidence intervals calculated where appropriate for each comparison.

Visual inspection using boxplots and histograms, followed by Shapiro–Wilk statistical tests, revealed approximate normality. A one-way repeated measures ANOVA was used to detect any differences in PAFAS, SDQ, and CYRM subscale scores across three measured intervention time points (pre, post, and follow-up) for the matched sample of 40 families who participated in this study. Bonferroni post hoc tests were performed to identify specific group differences. If data was not normal, non-parametric tests were used, including Friedman’s ANOVA with a Wilcoxon’s Signed Rank test for post hoc investigation. Mauchly’s test was used to check for sphericity (i.e., assumption that the variance of differences between all possible pairs of related groups are equal). In cases where the assumption was violated, Greenhouse–Geisser correction for violation was used.

Following the overall analysis of the three scales, a gendered sub-analysis was completed to determine group differences by both parent gender and participating child’s gender to understand any nuances in the data contributing to the findings. In previous studies [30,31,32], participants who had scores at baseline that indicated the most difficulty in SDQ (i.e., highest scores), high problems through PAFAS, or low resilience scores through CRYM-R were further analysed to determine potential effects on families having the most difficulties at the start (i.e., most-at-risk families).

## 3. Results

### 3.1. Recruitment, Follow-Up, and Erroneous Data

A total of 40 families were recruited and enrolled in the Strong Families programme in Cambodia. There was no loss to follow-up (t3). There were four errors in the data set, which were clarified with field officers. In two instances, the gender of the caregiver was not aligned with the relationship of the child (i.e., gender female and relationship with child’s father); this was clarified and aligned. In one case, the caregiver age was 3 years old in the dataset, and upon revisiting of logs, the data officer clarified it was an error and should read 30. Finally, there were two inputs in PAFAS t3 data that did not include one of the four choices available at the time of data collection. The field officer clarified that this was an error and rectified the correct inputs. There was no missing data; therefore, no imputation was necessary.

### 3.2. Demographics of Study Participants

The study sample included 40 families residing in rural communities in Cambodia, with most caregivers identifying as women and the majority living with a partner. Participating caregivers were all biological parents, generally in their mid-thirties, with limited access to higher education; nearly all had completed only primary education, as had their partners. Employment among caregivers was primarily home-based or informal, such as childcare, sewing, or small-scale trading, and a large proportion of partners were also engaged in unpaid or home-based work. Only a few participants reported travelling outside the village for employment. While most families were of Cambodian origin, some reported Lao heritage, and a small number identified as members of the Kavet Indigenous group. On average, caregivers had between two and three children. Participating children were aged 8 to 15, with a slightly higher number of boys than girls. A small portion of caregivers reported previous experience of war or conflict, though this was not common across the sample. There were no significant differences between the gender of caregivers across all the variables listed. Table 1 presents the demographic characteristics of Strong Families study participants in Cambodia.

### 3.3. Parenting and Family Adjustment Skills (PAFAS)

The findings reveal significant changes in the parenting practice subscales of parental consistency, coercive parenting, positive encouragement, and parent–child relationship, with substantial reductions observed from baseline to follow-up assessments. Additionally, analyses of family adjustment scores also show significant improvement across parental adjustment, family relationships, and parental teamwork subscales. This pattern, supported by strong effect sizes, suggests the positive influence of the intervention on familial dynamics and parental functioning. Table 2 outlines the mean PAFAS scores over time, overall, while also providing findings for families above and below the 75th percentile in the subcategories at baseline.

The most significant decrease from baseline (T1) to follow-up (T3) was for participants with scores above the 75th percentile at baseline across all the subscales, compared to the overall sample and the participants who scored below the 75th percentile.

### 3.4. Strengths and Difficulties Questionnaire (SDQ)

The total difficulty score decreased significantly (*p* = 0.0010) from 20.68 at baseline to 16.55 at follow-up, supported by a moderate effect size. The emotional and conduct problem scales also decreased while the prosocial scale significantly increased over time, trending in the right direction. Conversely, the peer problem scale significantly increased from pre to post and pre to follow-up, trending in the opposite intended direction. There was no significant difference over time in the overall hyperactivity scale.

Results by gender of the child participating in the study show that the total difficulty scale was significantly different for the girls but not the boys pre to post and pre to follow-up. Although there was no significant difference over time in overall hyperactivity, there was a significant improvement between pre and follow-up and again post and follow-up, while there was no significant difference for the boys. Participating families that started higher at baseline (above 75th percentile score) revealed an improvement in hyperactivity scores from baseline to post-intervention. Table 3 outlines the mean SDQ scores over time and by gender of participating children.

SDQ scores by gender of caregiver reveal that female caregivers experienced significant reductions in all the subscales except for hyperactivity, which had no significant findings, and peer problems, which significantly increased over time (Table 4). Male caregivers had significant improvements only in the prosocial scale and peer problem scale from pre to follow-up.

### 3.5. Child and Youth Resilience Measure (CYRM-R)

**Figure 2:**
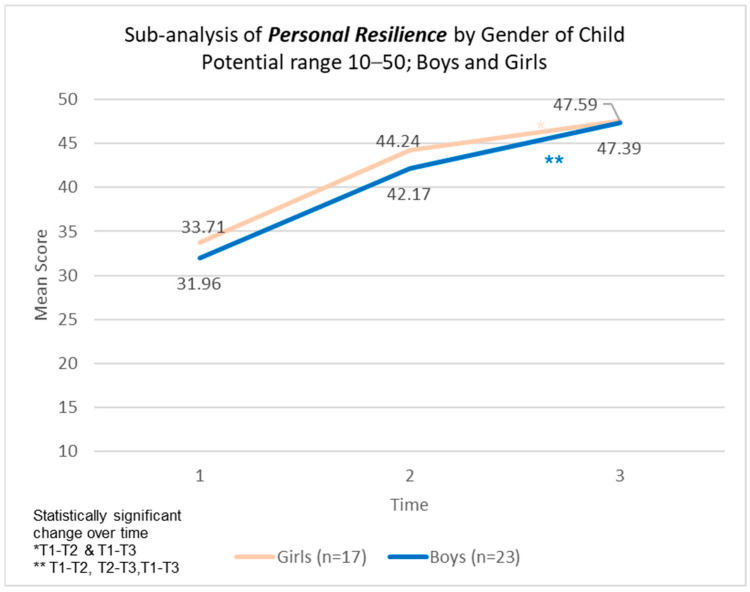
Personal resilience scores by gender of child participating in the programme.

**Figure 3:**
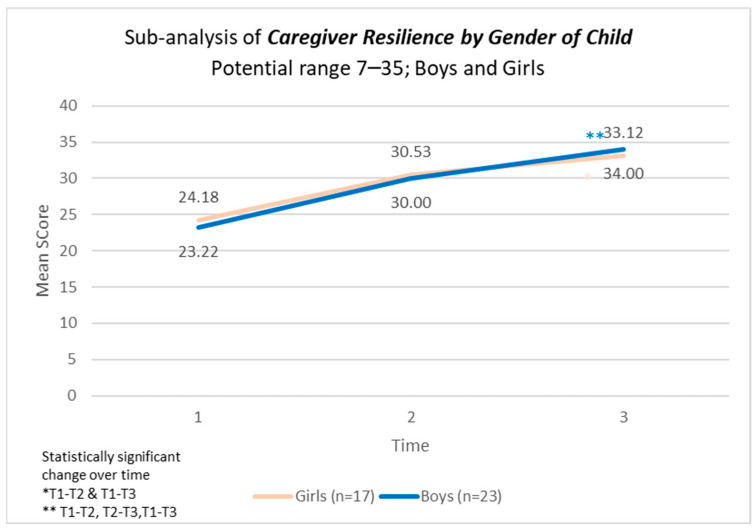
Caregiver resilience scores by gender of child participating in the programme.

**Figure 4:**
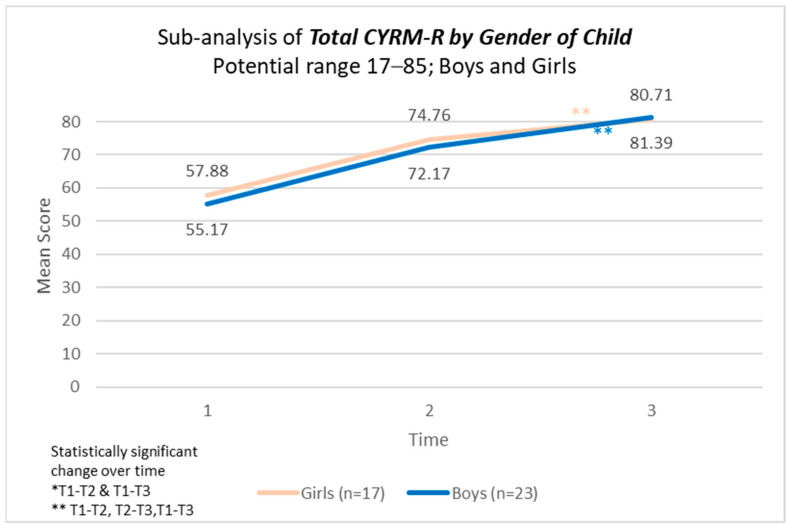
Total CYRM-R scores by gender of child participating in the programme.

There were statistically significant changes, supported by strong effect sizes, over time in the model across personal resilience, caregiver resilience, and total CYRM-resilience score across all three time points (Table 5). No statistically significant difference was found in CYRNM-R scores by gender of the child (Figure 2, Figure 3 and Figure 4).

## 4. Discussion

### 4.1. Overall Effect of the Strong Families Programme

This study builds on findings from earlier single-arm pilot studies of the Strong Families programme conducted in Afghanistan, Iran, and Serbia [30,31,32]. These prior studies demonstrated the programme’s feasibility and effectiveness in enhancing mental health, parenting practices, and family adjustment among families facing adversity. The findings of this study align with previous results, further reinforcing the programme’s impact—this time within the context of Cambodia, a lower-middle-income country in Southeast Asia. The findings suggest that the Strong Families programme was feasible to implement in a resource-limited setting. Facilitator training, recruitment, implementation, and retention rates within the programme were notably high and demonstrated practical feasibility. Despite the light design of the intervention, results indicated improved parenting skills, youth mental health, and adolescent resilience. The results focused on the short-term impact of the programme, as per its designed logical framework, similar to the effects found in the implementation in other LMICs, such as Afghanistan [31].

Furthermore, the average age of participating children from each family was higher than that of non-participating children, indicating a potential return on investment, as the intervention has scope to benefit the younger children in the families as well, once the caregivers have mastered the parenting components. Additionally, while all children experienced positive effects, those with lower baseline scores at T1 benefited the most. This was unsurprising as a similar trend was seen in previous Strong Families evaluations [30,31,32]. Thus, the various components of the programme, such as improving interactions between caregivers and their children, providing opportunities to learn and practice positive communication, learning stress management strategies, and engaging with behaviour management strategies, lead to the intended short-term results and potentially promote healthy psychosocial and physical development of children.

The effect sizes observed in our study—particularly in domains such as coercive parenting (*ηp*^2^ = 0.76) and prosocial behaviour (*ηp*^2^ = 0.79)—are comparable to those found in the Afghan pilot study, where similar large effects were observed across parenting outcomes (*ηp*^2^ ≈ 0.70–0.80) [31]. This consistency suggests that Strong Families has the potential to achieve robust improvements in caregiver and child outcomes across culturally diverse LMIC settings.

### 4.2. Effect of Strong Families on Parenting and Family Adjustment

Using the PAFAS, we evaluated improvements in parenting and family functioning skills, which serve as either protective or risk factors for children’s emotional and behavioural well-being. The findings suggest that the Strong Families programme enhances these areas by fostering positive and prosocial behaviour in children (e.g., reinforcing praise through “Using Love and Limits”), promoting reciprocal warmth and understanding within the family, equipping parents with tools to manage stress and anxiety, encouraging rule-setting to maintain a conflict-free home environment, and strengthening caregiver collaboration and support [42].

Since specific cut-off points could not have been established to predict future risk behaviour or to indicate the need for clinical interventions, we set the cut-off for our sub-analysis at the 75th percentile across each domain. Caregivers receiving the Strong Families programme indicated a significant improvement on all four parenting subscales and on all the family adjustment subscales. Further analysis of the most “at risk families” provided promising results indicating significant improvements were most evident in this group. This was further supported by higher effect sizes than in the overall changes in scores. These results align with findings from the Serbian trial, where effect sizes for parenting skills in high-risk families were similarly strong (*ηp*^2^ > 0.60), reinforcing the programme’s capacity to support families with the highest baseline difficulties [32]. In our Cambodia pilot, the greatest gains were also seen among those initially scoring in the top quartile of risk. This finding was very welcomed, though the researchers recognise that further research that utilises baseline assessments of presenting problems might be useful to explore if caregivers who score higher for challenges might be more receptive and amenable to parenting advice than those with lower scores. Nevertheless, although Strong Families is primarily a prevention intervention not specifically designed for families already facing mental or behavioural challenges, it is valuable to have further evidence that the programme can target and affect those at higher-risk of adverse parenting and adjustment skills.

From the results of this study, we can conclude that the intended short-term impact of the Strong Families programme regarding “Improved caregiver confidence in family management skills” and “Improved caregiving in parenting skills” was reached, as outlined in the logic model (Figure 1).

A long-term follow-up of the cohort is recommended to substantiate the long-term effects of the programme. According to the existing literature, the observed indicators suggest a trajectory towards the programme’s desired long-term outcomes. These include reductions in substance use, violence, and risky behaviours, as well as improved mental health of both caregivers and children [15,18].

### 4.3. Effect of Strong Families on Children’s Behaviour

When it comes to the SDQ scale, the total difficulty score decreased significantly from pre- to post-intervention. It is also worth noting the encouraging findings when looking specifically at the subscales, with the emotional and conduct problem scales decreasing while the prosocial scale significantly increasing over time, trending in the right direction. Our results suggest that the intervention led to reductions in child behavioural and emotional difficulties, supporting previous research that even very light-touch interventions can bring about change in families in low-resource settings [30,31,32,49,50]. These outcomes correspond to research demonstrating the positive relationship between improving parenting and resultant positive outcomes for children, such as their improved emotional and behavioural regulation and socialisation in conflict [51,52].

In several cases, the most significant improvements were observed at the final assessment, conducted six weeks post-intervention. This pattern mirrors findings from randomised controlled trials (RCTs) of short parenting programmes in both Panama and Serbia, where intervention effects on parental reports of behavioural difficulties were moderate immediately post-programme but became more pronounced at the final follow-up [26,32]. One possible explanation for this delayed impact is that psychological changes facilitated by the programme, such as increased parental confidence and enhanced problem-solving abilities, take time to translate into tangible behavioural shifts in both caregivers and children. With continued practice and reinforcement, these improvements may become more evident over time. Further research investigating these delayed but compounding effects would be valuable.

Conversely, the peer problem scale significantly increased from pre to post and pre to follow-up, trending in the opposite intended direction. This effect was seen in a previous pilot [32]. Addressing peer problems does not feature as part of the logic model for Strong Families, but through the piloting globally of Strong Families, it has become clear that it would be beneficial to address this topic in future interventions. A Peer Pressure “add on” module has been developed since this pilot and is now implemented alongside the original Strong Families manual, with evaluations on its effectiveness underway.

In addition, hyperactivity was significantly reduced among girls but not boys. In previous evaluations of Strong Families, we had not seen this trend. Possible explanations might be that the cultural interpretation of the questions in this subscale might hold different meanings for boys and girls. A re-evaluation of this question is planned for an upcoming trial.

### 4.4. Effect of Strong Families on Child Resilience

In the CYRM scale, personal resilience encompasses both intrapersonal and interpersonal factors shaped by an individual’s social ecology to enhance their capacity for resilience. Meanwhile, caregiver resilience reflects the supportive relationships formed with a primary caregiver or family members [45]. In this study, children in Cambodia had an average baseline score of approximately 32 for personal resilience and 23 for caregiver resilience. When compared to children from 14 other countries with similar age and gender distributions, Cambodian children in our sample appeared to score on the lower end of the scale, with global averages around 40 and 28 points, respectively [53]. Given this context, the findings of our study are particularly significant, demonstrating statistically meaningful improvements over time across both subscales—personal resilience and caregiver resilience—as well as the overall CYRM-Resilience Score, with strong effect sizes. Resilience represents an individual’s ability to effectively adapt and thrive despite significant life challenges. Previous research suggests that the mechanisms underpinning resilience in response to everyday stressors are similar to those required for coping in post-disaster situations [54,55]. A light-touch, five-session intervention that can significantly improve resilience has the potential to have compounding effects.

Furthermore, a significant body of research that spans various countries and examines common elements within adolescent resilience networks identifies caregiver support as a crucial factor for fostering adolescent resilience. This finding is supported by a comparative study involving 18,914 adolescents across 14 countries, where caregiver support showed the most substantial positive correlations with other resilience resources [53]. This evidence underscores the importance of interventions designed to enhance caregiver support skills as a strategy for building resilience in youth.

### 4.5. Limitations and Strengths

Our study has several limitations to consider. The sample precludes the generalizability of findings beyond families in Cambodia. Secondly, while our programme and recruitment efforts aimed for universal participation, the sample primarily consisted of mothers. The underrepresentation of fathers in parenting research and programme implementation is a well-documented challenge in the literature, particularly in underserved settings and LMICs. Thus, this study’s findings cannot be generalised to the male caregiver population. Further research is being carried out looking to explore the role of fathers/male caregivers in family skills programmes and investigating the potential of including both caregivers in such programmes. Thirdly, of added value and interest to us is to explore the long-term follow-up of participants, further to the short-term six-week post-intervention that was carried out in this study. Finally, while the evaluation tools used in this study were either previously translated into Khmer (SDQ) or adapted through translation and back-translation procedures in-country (PAFAS), formal psychometric validation of these instruments was not conducted in the Cambodian context, representing a methodological limitation and an area for future research.

Despite these limitations, our study has several strengths. Our results are encouraging, demonstrating the programme’s effectiveness across yet another setting. This highlights the value of Strong Families, particularly in resource-limited environments where facilitator time and training may be constrained. As demonstrated in this study and our previous ones, facilitators do not require formal education or extensive training, so the programme can be scaled up with motivated individuals who have direct access to families. The family skills programmes have long-term sustainability potential through routine family services, as the investment in facilitator training is minimal compared to the lasting benefits for families. Family skills programmes have been widely recognised as effective primary prevention strategies, offering a more cost-effective approach than treating mental health or substance use disorders [21,56,57].

Estimated cost-effective interventions for key mental, neurological, and substance use disorders in LMICs are approximately USD 3–4 per capita annually [57]. These conditions often take a chronic or disabling trajectory, leading to additional treatment costs, yet less than 1% of health expenditures are allocated to this population.

Family skills interventions have been widely endorsed for primary prevention, including reducing drug use and interpersonal violence—especially against children—by key global organisations such as the UNODC-WHO International Standards on Drug Use Prevention [21], the WHO Violence Prevention Alliance for Youth Violence Prevention [23], and the INSPIRE Interagency Initiative to end violence against children [22]. Evidence suggests that investing in these interventions is both effective and cost-efficient [21], as well as advancing several targets of the Sustainable Development Goals. Recognising parenting as a crucial determinant in improving child outcomes and mitigating risks associated with low-resource environments is essential. Likewise, these findings highlight the role of parents in strengthening family engagement, enhancing caregiving, fostering communication, and building reciprocal support systems, even in the face of adversity. The findings of this pilot study align with these objectives.

## 5. Conclusions

Family mental health and well-being should receive the same level of priority as access to medical care, sanitation, and clean water is essential for effectively supporting families in LMICs. Addressing both collective and individual mental well-being is critical in fostering resilience and positive family dynamics. Findings from our Cambodia pilot study demonstrate that implementing a family skills programme is both feasible and beneficial in resource-limited and high-stress environments. The Strong Families programme, designed as a light-touch intervention, can be deliverable by both laypersons and trained facilitators. Further research is needed to evaluate its long-term impact on LMICs in Southeast Asia and to conduct comparative assessments of children who have participated in the programme versus those who have not. These findings align with our studies implementing the Strong Families programme in other countries, reinforcing its cross-cultural adaptability and effectiveness despite variations in service provision and local contexts. By demonstrating the feasibility of these interventions, this study can inform policymakers and encourage the integration of family skills programmes into national strategies aimed at reducing adverse health and social outcomes. Such initiatives enhance familial support structures, enhancing the overall well-being and reliance of young people in challenged settings.

## Figures and Tables

**Table 1:** Demographic characteristics of study participants in the Cambodia Strong Families programme.

		Total (n = 40)	Female Caregivers (n = 33)	Male Caregivers (n = 3)
Variables		Mean (SD), % (n)	Mean (SD), % (n)	Mean (SD), % (n)
Age (in years)		35.725 (8.732)	34.64 (1.532)	40.86 (2.539)
Marital Status	Partnered (married or cohabitating)	39 (97.5%)	32 (97%)	7 (100%)
	Divorced or separated	1 (2.5%)	1 (3%)	0 (0%)
Caregiver Education	Caregiver primary education	37 (92.5%)	37 (94%)	6 (86%)
	Caregiver higher education	3 (7.5%)	2 (6%)	1 (14%)
Partner Education	Partner primary education	36 (90%)	29 (88%)	7 (100%)
	Partner higher education	4 (10%)	4 (12%)	0 (0%)
Employment Status Caregiver	Caregiver working FT	5 (12.5%)	4 (12%)	1 (14%)
	Caregiver working PT	2 (5%)	2 (6%)	0 (0%)
	Caregiver looking for work	1 (2.5%)	0 (0%)	1 (14%)
	Caregiver working at home paid	27 (67.5%)	22 (67%)	5 (71%)
	Caregiver not working	5 (12.5%)	5 (15%)	0 (0%)
Employment Status Partner	Partner working FT	4 (10%)	3 (9%)	1 (14%)
	Partner working PT	1 (2.5%)	1 (3%)	0 (0%)
	Partner looking for work	12 (30%)	9 (27%)	3 (43%)
	Partner working at home paid	20 (50%)	17 (51%)	3 (43%)
	Partner not working	3 (7.5%)	3 (9%)	0 (0%)
Travel for Work Outside Village	Caregiver travels	7 (17.5%)	7 (21%)	0 (0%)
	Caregiver does not travel	33 (82.5%)	26 (79%)	7 (100%)
	Partner travel outside village for work	7 (17.5%)	7 (21%)	0 (0%)
	Partner does not travel outside village for work	33 (82.5%)	26 (79%)	7 (100%)
Experience of War/Conflict	Experienced war/conflict	5 (12.5%)	3 (9%)	2 (29%)
	Did not experience war/conflict	35 (87.5%)	30 (91%)	5 (71%)
Ethnicity	Ka Bet ethnicity	6 (15%)	5 (15%)	1 (14%)
	Does not belong to ethnic group	34 (85%)	28 (85%)	6 (86%)
Characteristics of Children	Number of children	2.58 (0.984)	2.48 (0.972)	3 (1)
	Average age of child in the programme in yrs.	10.38 (2.676)	10.36 (2.643)	10.42857 (3.047)
Gender of Child	Girl	17 (42.5%)	15 (45.5%)	2 (28.6%)
	Boy	23 (57.5%)	18 (54.5%)	5 (71.4%)
Age by Gender of Child				
	Girl (n = 17)	9.76 (2.773)		
	Boy (n = 33)	10.83 (2.70)		

**Table 2:** Mean PAFAS scores over time overall and for families above and below the 75th percentile in each subcategory at baseline.

	Pre-Test Mean (SD) [Min–Max]	Post-Test Mean (SD) [Min–Max]	Follow-Up Mean (SD) [Min–Max]	Test Statistic (Repeated Measures ANOVA or Friedman’s χ^2^)		Pairwise Comparison
				F (df_time_, df_error_)/χ^2^(df)	*p*-Value	
PARENTING						
Parental Consistency [0–15]						
Overall	8.13	5.95	5.93	F(2,78) = 17.96	0.0000	b,c
n = 40	1.40	2.40	1.69	*ηp*^2^ = 0.49		
	[4–11]	[2–12]	[2–9]			
≥75 percentile @ T1 [score = 9–15]	9.44	6.31	5.56	F(2,30) = 23.90	0.0000	b,d
n = 16	0.63	2.57	1.63	*ηp*^2^ = 0.79		
	[9–12]	[2–12]	[3–9]			
<75 percentile@ T1 [score <9]	7.25	5.71	6.17	F(2,46) = 4.48	0.0167	b
n = 24	1.03	2.29	1.71	*ηp*^2^ = 0.30		
	[4–8]	[3–10]	[2–9]			
Coercive Parenting [0–15]						
Overall	8.13	4.00	2.33	F(2,78) = 59.76	0.0000	a,b,c
n = 40	2.61	2.80	2.76	*ηp*^2^ = 0.76		
	[3–14]	[0–15]	[0–12]			
≥75 percentile @ T1 [score = 9.75–15]	11.50	4.10	4.20	F(2,18) = 22.23	0.0000	b,d
n = 10	1.43	2.38	4.29	*ηp*^2^ = 0.86		
	[10–14]	[2–9]	[0–12]			
<75 percentile @ T1 [score < 9.75]	7.00	3.97	1.70	F(2,58) = 49.67	0.0000	b,c,d
n = 30	0.33	2.97	1.70	*ηp*^2^ = 0.79		
	[3–9]	[0–15]	[0–6]			
Positive Encouragement [0–9]						
Overall	2.60	1.00	0.33	F(2,78) = 27.05	0.0000	b,c
n = 40	1.91	1.34	0.92	*ηp*^2^ = 0.59		
	[0–7]	[0–5]	[0–3]			
≥75 percentile @ T1 [score 4–9]	4.79	0.93	0.64	F(2,26) = 44.87	0.0000	b,d
n = 14	1.19	1.27	1.28	*ηp*^2^ = 0.89		
	[4–7]	[0–4]	[0–3]			
<75 percentile @ T1 [score < 4]	1.42	1.04	0.15	F(2,50) = 10.73	0.0001	c,d
n = 26	0.90	1.40	0.61	*ηp*^2^ = 0.48		
	[0–3]	[0–5]	[0–3]			
Parent–Child Relationship [0–9]						
Overall	4.08	1.48	0.60	χ^2^(2) = 30.97	0.0000	b,c,d
n = 40	2.76	2.18	1.69	*W* = 0.39		
	[0–10]	[0–8]	[0–9]			
≥75 percentile @ T1 [score = 6–9]	7.50	1.00	1.25	χ^2^(2) = 17.33	0.0002	b,d
n = 12	1.31	1.35	2.70	*W* = 0.72		
	[6–10]	[0–3]	[0–9]			
<75 percentile @ T1 [score < 6]	2.61	1.68	0.32	χ^2^(2) = 17.23	0.0020	c,d
n = 28	1.69	2.45	0.94	*W* = 0.31		
	[0–5]	[0–8]	[0–3]			
FAMILY ADJUSTMENT						
Parental Adjustment [0–15]						
Overall	5.63	4.45	1.75	F(2,78) = 33.03	0.0000	c,d
n = 40	1.85	2.47	2.31	*ηp*^2^ = 0.6413		
	[0–10]	[0–9]	[0–9]			
≥75 percentile @ T1 [score = 6.75–15]	7.90	4.50	1.50	χ^2^(2) = 10.76	0.0046	b,c,d
n = 10	1.20	2.88	2.92	*W* = 0.54		
	[7–10]	[0–9]	[0–9]			
<75 percentile @ T1 [score < 6.75]	4.87	4.43	1.83	χ^2^(2) = 25.45	0.0000	c,d
n = 30	1.33	2.37	2.12	*W* = 0.42		
	[0–6]	[0–8]	[0–6]			
Family Relationships [0–12]						
Overall	4.43	2.13	0.68	χ^2^(2) = 45.62	0.0000	b,c,d
n = 40	1.92	1.99	1.70	*W* = 0.57		
	[0–9]	[0–8]	[0–9]			
≥75 percentile @ T1 [score = 5.75–12]	6.80	2.30	0.90	χ^2^(2) = 15.08	0.0005	b,c,d
n = 10	0.33	0.75	0.41	*W* = 0.75		
	[6–9]	[0–8]	[0–3]			
<75 percentile @ T1 [score < 5.75]	3.63	2.07	0.60	χ^2^(2) = 30.91	0.0000	b,c,d
n = 30	1.43	1.89	1.83	*W* = 0.52		
	[0–5]	[0–6]	[0–9]			
Parental Teamwork [0–9]						
Overall	2.68	1.63	0.23	χ^2^(2) = 31.24	0.0000	b,c,d
n = 40	1.99	1.75	0.70	*W* = 0.39		
	[0–6]	[0–5]	[0–3]			
≥75 percentile @ T1 [score = 4–9]	4.75	2.25	0.125	F(2,30) = 45.53	0.0000	b,c,d
n = 16	0.194	0.552	0.085	*ηp*^2^ = 0.88		
	[4–6]	[0–5]	[0–1]			
<75 percentile @ T1 [score < 4]	1.29	1.21	0.29	F(2,46) = 7.39	0.0019	c,d
n = 24	1.16	1.25	0.86	*ηp*^2^ = 0.41		
	[0–3]	[0–3]	[0–3]			

Statistically significant (*p* < 0.05); SD: standard deviation; a data not normally distributed, non-parametrical tests used for all statistics involving this group; b significant difference between t1 and t2; c significant difference between t2 and t3; d significant difference between t1 and t3. Effect size calculations: *ηp*^2^ indicates partial eta squared effect size for repeated measures ANOVA (F), calculated ${\eta p}^{2}=\frac{F\times \left(k-1\right)}{F\times \left(k-1\right)+(n-k)}$; W indicates Kendall’s *W* effect size for Friedman’s ANOVA (χ^2^), calculated as Kendall’s *W* $=\frac{\chi^{2}}{n(k-1)}$, where n = sample size (40), k = number of time points (3). Effect sizes are interpreted as follows—partial eta squared (*ηp*^2^): small ≥ 0.01, moderate ≥ 0.06, large ≥ 0.14; Kendall’s W: small ≥ 0.10, moderate ≥ 0.30, large ≥ 0.50.

**Table 3:** Mean SDQ score over time overall and for families above and below the 75th percentile in hyperactivity at baseline and by gender of participating child.

SDQ	Pre-Test Mean (SD) [Min–Max]	Post-Test Mean (SD) [Min–Max]	Follow-Up Mean (SD) [Min–Max]	Test Statistic (Repeated Measures ANOVA or Friedman’s χ^2^)		Pairwise Comparison
				F (df_time_, df_error_)/χ^2^(df)	*p*-Value	
Emotional problem scale [0–10]						
**Overall**	5.60	4.30	2.375	χ^2^(2) = 21.17	0.0000	b,c,d
	1.65	2.65	2.87	*W* = 0.26		
	[2–8]	[0–9]	[0–10]			
**Girls (n = 17)**	5.412	4.471	2.000	χ^2^(2) = 9.71	0.0078	c,d
	1.326	2.375	2.622	*W* = 0.29		
	[3–8]	[0–8]	[0–10]			
**Boys (n = 23)**	5.739	4.174	2.652	χ^2^(2) = 11.88	0.0026	b,c,d
	1.864	2.887	3.069	*W* = 0.26		
	[2–8]	[0–9]	[0–10]			
Conduct problem scale [0–10]						
**Overall**	4.33	3.38	2.68	F(2,78) = 11.35	0.0000	b,d
	1.85	1.88	1.61	*ηp*^2^ = 0.38		
	[1–9]	[0–9]	[1–10]			
**Girls (n = 17)**	4.53	3.35	2.65	F(2,32) = 11.35	0.0005	b,d
	1.70	1.50	1.17	*ηp*^2^ = 0.62		
	[2–7]	[2–6]	[2–6]			
**Boys (n = 23)**	4.17	3.39	2.70	F(2,44) = 4.03	0.0280	d
	1.97	2.15	1.89	*ηp*^2^ = 0.29		
	[1–9]	[0–9]	[1–10]			
Hyperactivity scale [0–10]						
**Overall**	5.28	5.2 (*)	4.95	χ^2^(2) = 2.43	0.2974	-
	1.74	1.94	2.28	*W* = 0.03		
	[2–9]	[2–9]	[2–10]			
**Girls (n = 17)**	5.24	5.41	3.82	χ^2^(2) = 10.13	0.0063	c,d
	1.56	1.80	1.24	*W* = 0.30		
	[2–8]	[3–9]	[2–6]			
**Boys (n = 23)**	5.30	5.04	5.78	χ^2^(2) = 0.53	0.7691	-
	1.89	2.06	2.52	*W* = 0.01		
	[3–9]	[2–9]	[2–10]			
>=75 percentile @ T1 [score = 6+]						
**n = 18**	6.889	5.556	5.667	χ^2^(2) = 7.37	0.0251	b
	0.963	2.064	2.787	*W* = 0.20		
	[6–9]	[2–9]	[2–10]			
<75 percentile @ T1 [score <6]						
**n = 22**	3.955	4.909	4.364	F(2,42) = 2.43	0.1004	-
	0.899	1.823	1.590	*ηp*^2^ = 0.20		
	[2–5]	[2–9]	[2–8]			
Peer problem scale [0–10]						
**Overall**	5.48	6.73	6.55	F(2,78) = 7.34	0.0012	b,d
	1.57	1.68	1.81	*ηp*^2^ = 0.28		
	[3–10]	[2–10]	[2–10]			
**Girls (n = 17)**	5.47	7.00	6.00	F(2,32) = 5.11	0.0110	b
	1.46	1.41	1.62	*ηp*^2^ = 0.42		
	[3–8]	[4–10]	[2–8]			
**Boys (n = 23)**	5.48	6.52	6.96	F(2,44) = 4.86	0.0124	d
	1.68	1.86	1.87	*ηp*^2^ = 0.33		
	[3–10]	[2–9]	[4–10]			
Prosocial scale [10–0]						
**Overall**	5.60	8.15	9.45	F(2,78) = 69.93	0.0000	b,c,d
	1.30	1.86	1.04	*ηp*^2^ = 0.79		
	[3–9]	[4–10]	[6–10]			
**Girls (n = 17)**	5.53	8.59	9.35	F(2,32) = 36.61	0.0000	b,d
	1.23	1.37	1.17	*ηp*^2^ = 0.84		
	[4–8]	[6–10]	[6–10]			
**Boys (n = 23)**	5.65	7.83	9.52	F(2,44) = 36.29	0.0000	b,c,d
	1.37	2.12	0.95	*ηp*^2^ = 0.78		
	[3–9]	[4–10]	[7–10]			
Total difficulty scale [0–40]						
**Overall**	20.68	19.60	16.55	F(2,78) = 7.58	0.0010	c,d
	5.15	6.14	5.73	*ηp*^2^ = 0.29		
	[10–29]	[9–35]	[10–35]			
**Girls (n = 17)**	20.65	20.24	14.47	F(2,32) = 12.40	0.0001	c,d
	4.49	5.36	3.10	*ηp*^2^ = 0.64		
	[13–29]	[12–30]	[10–24]			
**Boys (n = 23)**	20.70	19.13	18.09	F(2,44) = 1.40	0.2568	-
	5.68	6.74	6.73	*ηp*^2^ = 0.12		
	[10–29]	[9–35]	[10–35]			

Statistically significant (*p* < 0.05); SD: standard deviation; b significant difference between t1 and t2; c significant difference between t2 and t3; d significant difference between t1 and t3. Effect size calculations *ηp*^2^ indicates partial eta squared effect size for repeated measures ANOVA (F), calculated ${\eta p}^{2}=\frac{F\times \left(k-1\right)}{F\times \left(k-1\right)+(n-k)}$; W indicates Kendall’s *W* effect size for Friedman’s ANOVA (χ^2^), calculated as Kendall’s *W* $=\frac{\chi^{2}}{n(k-1)}$, where n = sample size (40), k = number of time points (3). Effect sizes are interpreted as follows—partial eta squared (*ηp*^2^): small ≥ 0.01, moderate ≥ 0.06, large ≥ 0.14; Kendall’s W: small ≥ 0.10, moderate ≥ 0.30, large ≥ 0.50.

**Table 4:** Mean SDQ score over time by gender of caregiver.

	Pre-Test Mean (SD) [Min–Max]	Post-Test Mean (SD) [Min–Max]	Follow-Up Mean (SD) [Min–Max]	Test Statistic (Repeated Measures ANOVA or Friedman’s χ^2^)		Pairwise Comparison
				F (df ^comparison time^, df^error^)/χ^2^(df)	*p*-Value	
Total difficulty scale [0–40]						
Female caregivers (n = 33)	20.42	19.52	16.12	F(2,64) = 6.56	0.0026	c,d
	5.21	5.90	5.68	*ηp*^2^ = 0.30		
	[10–29]	[9–30]	[10–35]			
Male caregivers (n = 7)	21.86	20.00	18.57	F(2,12) = 1.05	0.3813	-
	5.01	7.70	5.94	*ηp*^2^ = 0.34		
	[14–27]	[14–35]	[12–26]			
Emotional problem scale [0–10]						
Female caregivers (n = 33)	5.45	4.30	2.30	F(2,64) = 17.17	0.0000	c,d
	1.62	2.54	3.04	*ηp*^2^ = 0.53		
	[2–8]	[0–8]	[0–10]			
Male caregivers (n = 7)	6.29	4.29	2.71	χ^2^(2) = 4.92	0.0853	d
	1.70	3.35	2.06	*W* = 0.35		
	[4–8]	[0–9]	[0–5]			
Conduct problem scale [0–10]						
Female caregivers (n = 33)	4.45	3.30	2.79	F(2,64) = 10.03	0.0002	b,d
	1.95	1.74	1.73	*ηp*^2^ = 0.40		
	[1–9]	[0–7]	[2–10]			
Male caregivers (n = 7)	3.71	3.71	2.14	F(2,12) = 2.27	0.1460	-
	1.11	2.56	0.69	*ηp*^2^ = 0.53		
	[2–5]	[2–9]	[1–3]			
Hyperactivity scale [0–10]						
Female caregivers (n = 33)	5.09	5.27	4.67	F(2,64) = 0.93	0.3932	-
	1.65	2.00	2.10	*ηp*^2^ = 0.06		
	[2–8]	[2–9]	[2–10]			
Male caregivers (n = 7)	6.14	4.86	6.29	χ^2^(2) = 1.83	0.4013	-
	2.04	1.68	2.75	*W* = 0.13		
	[3–9]	[3–8]	[4–10]			
Peer problem scale [0–10]						
Female caregivers (n = 33)	5.42	6.64	6.36	F(2,64) = 5.23	0.0079	b,d
	1.66	1.65	1.76	*ηp*^2^ = 0.26		
	[3–10]	[2–10]	[2–10]			
Male caregivers (n = 7)	5.71	7.14	7.43	χ^2^(2) = 5.56	0.0622	d
	1.11	1.86	1.90	*W* = 0.40		
	[4–7]	[4–9]	[5–10]			
Prosocial scale [10–0]						
Female caregivers (n = 33)	5.55	8.24	9.39	F(2,64) = 59.50	0.0000	b,c,d
	1.35	1.84	1.09	*ηp*^2^ = 0.80		
	[3–9]	[4–10]	[6–10]			
Male caregivers (n = 7)	5.86	7.71	9.71	F(2,12) = 10.45	0.0024	d
	1.07	2.06	0.76	*ηp*^2^ = 0.84		
	[4–7]	[4–10]	[8–10]			

Statistically significant (*p* < 0.05); SD: standard deviation; b significant difference between t1 and t2; c significant difference between t2 and t3; d significant difference between t1 and t3. Effect size calculations *ηp*^2^ indicates partial eta squared effect size for repeated measures ANOVA (F), calculated ${\eta p}^{2}=\frac{F\times \left(k-1\right)}{F\times \left(k-1\right)+(n-k)}$; W indicates Kendall’s *W* effect size for Friedman’s ANOVA (χ^2^), calculated as Kendall’s *W* $=\frac{\chi^{2}}{n(k-1)}$, where n = sample size (40), k = number of time points (3). Effect sizes are interpreted as follows—partial eta squared (ηp^2^): small ≥ 0.01, moderate ≥ 0.06, large ≥ 0.14; Kendall’s W: small ≥ 0.10, moderate ≥ 0.30, large ≥ 0.50.

**Table 5:** Mean scores for CYRM-R over time.

Pre-Test Mean (SD) [Min–Max]	Post-Test Mean (SD) [Min–Max]	Follow-Up Mean (SD) [Min–Max]	Test Statistic (Repeated Measures ANOVA or Friedman’s χ^2^)		Pairwise Comparison
			χ^2^(df)	*p*-Value	
Personal Resilience [10–15]; n = 40					
32.70	43.05	47.48	χ^2^(2) = 62.42	0.0000	b,c,d
5.33	5.38	3.79	*W* = 0.78		
[24–42]	[28–50]	[30–50]			
Caregiver Resilience [7–35]; n = 40					
23.63	30.23	33.63	χ^2^(2) = 61.83	0.0000	b,c,d
5.36	3.97	2.54	*W* = 0.77		
[12–34]	[19–35]	[21–35]			
Total Resilience [17–85]; n = 40					
56.3	73.3	81.1	χ^2^(2) = 64.67	0.0000	b,c,d
10.0	8.8	5.8	*W* = 0.81		
[37–72]	[47–82]	[51–85]			

Statistically significant (*p* < 0.05); SD: standard deviation; b significant difference between t1 and t2; c significant difference between t2 and t3; d significant difference between t1 and t3. Effect size calculations: W indicates Kendall’s *W* effect size for Friedman’s ANOVA (χ^2^), calculated as Kendall’s *W* $\frac{\chi^{2}}{n(k-1)}$, where n = sample size (40), k = number of time points (3). Effect sizes are interpreted as follows: Kendall’s W: small ≥ 0.10, moderate ≥ 0.30, large ≥ 0.50.

## Data Availability

Data is contained within the article.
